# Endothelial microparticles are increased in congenital heart diseases and contribute to endothelial dysfunction

**DOI:** 10.1186/s12967-016-1087-2

**Published:** 2017-01-04

**Authors:** Ze-Bang Lin, Hong-Bo Ci, Yan Li, Tian-Pu Cheng, Dong-Hong Liu, Yan-Sheng Wang, Jun Xu, Hao-Xiang Yuan, Hua-Ming Li, Jing Chen, Li Zhou, Zhi-Ping Wang, Xi Zhang, Zhi-Jun Ou, Jing-Song Ou

**Affiliations:** 1 Division of Cardiac Surgery, The First Affiliated Hospital of Sun Yat-sen University, 58 Zhong Shan Er Road, Guangzhou, 510080 People’s Republic of China; 2 Department of Ultrasound, The First Affiliated Hospital of Sun Yat-sen University, Guangzhou, 510080 People’s Republic of China; 3 Division of Hypertension and Vascular Diseases, The First Affiliated Hospital of Sun Yat-sen University, Guangzhou, 510080 People’s Republic of China; 4 The Key Laboratory of Assisted Circulation, Ministry of Health, Guangzhou, 510080 People’s Republic of China; 5 National and Guangdong Province Joint Engineering Laboratory for Diagnosis and Treatment of Vascular Diseases, Guangzhou, 510080 People’s Republic of China; 6 Guangdong Provincial Key Laboratory of Brain Function and Disease, Guangzhou, 510080 People’s Republic of China; 7 State Key Laboratory of Respiratory Disease, Guangzhou, 510080 People’s Republic of China; 8 Guangzhou Institute of Respiratory Disease, Guangzhou, 510080 People’s Republic of China; 9 The First Affiliated Hospital of Guangzhou Medical University Guangzhou, Guangzhou, 510120 People’s Republic of China

**Keywords:** Endothelial microparticles, Congenital heart disease, Inflammation, Endothelial nitric oxide synthase, P38 MAPK pathway

## Abstract

**Background:**

We previously demonstrated that endothelial microparticles (EMPs) are increased in mitral valve diseases and impair valvular endothelial cell function. Perioperative systemic inflammation is an important risk factor and complication of cardiac surgery. In this study, we investigate whether EMPs increase in congenital heart diseases to promote inflammation and endothelial dysfunction.

**Methods:**

The level of plasma EMPs in 20 patients with atrial septal defect (ASD), 23 patients with ventricular septal defect (VSD), and 30 healthy subjects were analyzed by flow cytometry. EMPs generated from human umbilical vascular endothelial cells (HUVECs) were injected into C57BL6 mice, or cultured with HUVECs without or with siRNAs targeting P38 MAPK. The expression and/or phosphorylation of endothelial nitric oxide synthase (eNOS), P38 MAPK, and caveolin-1 in mouse heart and/or in cultured HUVECs were determined. We evaluated generation of nitric oxide (NO) in mouse hearts, and levels of tumor necrosis factor-α (TNF-α) and interleukin-6 (IL-6) in cultured HUVECs and in mice.

**Results:**

EMPs were significantly elevated in patients with ASD and VSD, especially in those with pulmonary hypertension when compared with controls. EMPs increased caveolin-1 expression and P38 MAPK phosphorylation and decreased eNOS phosphorylation and NO production in mouse hearts. EMPs stimulated P38 MAPK expression, TNF-α and IL-6 production, which were all inhibited by siRNAs targeting P38 MAPK in cultured HUVECs.

**Conclusions:**

EMPs were increased in adult patients with congenital heart diseases and may contribute to increased inflammation leading to endothelial dysfunction via P38 MAPK-dependent pathways. This novel data provides a potential therapeutic target to address important complications of surgery of congenial heart disease.

**Electronic supplementary material:**

The online version of this article (doi:10.1186/s12967-016-1087-2) contains supplementary material, which is available to authorized users.

## Background

Recently, we demonstrated that circulating microparticles in patients with valvular heart disease impaired endothelium dependent vasodilation by uncoupling and inhibiting endothelial nitric oxide synthase (eNOS) [1]. Microparticles are generated from a variety of sources including endothelial cells, platelets, T cells, etc. We previously showed that microparticles generated from endothelial cells induce acute lung injury, inhibit angiogenesis, and impair vasodilation [2, 3]. We also demonstrated that the number of plasma endothelial microparticles (EMPs) is significantly higher in patients with mitral valve diseases leading to impairment of human mitral valve endothelial cell function [4]. In proteomics studies, we found that EMPs contain hundreds of proteins, some of which may impair vasodilation, induce lung injury and/or activate inflammation [5, 6]. Thus, EMPs are considered both a marker and a mediator of endothelial injury. Previously Amabile reported that EMP levels are correlated with pulmonary arterial pressure in adults with primary and secondary pulmonary arterial hypertension [7]. However, Samadja showed no significant difference in EMPs levels between irreversible and reversible pulmonary arterial hypertension in children with congenital heart disease [8]. Thus, whether EMPs are consistently elevated in all congenital heart diseases remains unclear.

Perioperative systemic inflammation is a major risk factor and complication of cardiac surgery. Cardiac surgery with cardiopulmonary bypass (CPB) results in systemic inflammatory responses that contribute to instability of circulation post-operatively [9, 10]. Preventza et al. reported that CPB time is related to mortality after surgery [11]. Although we previously demonstrated that EMPs contain proteins that can induce inflammation, the impact and the mechanisms of action of EMPs to promote an inflammatory response are not fully understood. In this study, we investigated, for the first time, whether EMPs increased in two common congenital heart diseases, atrial septal defect (ASD) and ventricular septal defect (VSD), and the impact and the mechanisms of action of EMPs to promote inflammation and subsequent endothelial dysfunction.

## Methods

Please refer to the Additional file 1 for additional details on the methodology.

### Study population

20 patients diagnosed with ASD, 23 patients with VSD, and 30 age-matched healthy volunteer control subjects were recruited at The First Affiliated Hospital of Sun Yat-sen University. Patients with diseases which may increase EMPs level, including coronary heart disease, hypertension, infectious disease, severe trauma, antibiotic therapy, lupus anticoagulant, multiple sclerosis, renal failure, rheumatic diseases in acute stage, and valvular heart disease were explicitly excluded. Healthy volunteers below 18 years old and those who abused alcohol and/or heavy smokers were excluded. This study was approved by the Ethics Committee of The First Affiliated Hospital, Sun Yat-sen University. Informed consent was obtained from all subjects enrolled in this study. Clinical characteristics, doppler echocardiographic metrics (the definition and classification of pulmonary hypertension are referenced as in the 2014 Nice Pulmonary Hypertension Classification System) and operation data were collected (Table 1).

**Table 1:** Clinical characteristic and Doppler echocardiographic variables

Variables	Control group	Atrial septal defect group	Ventricular septal defect group
N	30	20	23
Gender (M/F)	12/18	7/13	10/13
Age (y)	39.87 ± 13.17	35.85 ± 14.12	38.30 ± 14.81
PH, N (%)	0	15 (75%)	6 (26.1%)
NYHA Class (II/III)	0	16/4	15/8
CPB time, min	–	66 ± 28	75 ± 23
Aortic cross clamp time, min	–	33 ± 24	39 ± 20
LAD, mm	29.93 ± 2.69	33.75 ± 5.11*	36.48 ± 9.98*
LVEDD, mm	45.903.16	39.75 ± 3.89*	51.78 ± 11.43*^#^
LVESD, mm	28.80 ± 2.72	25.55 ± 2.87*	31.64 ± 8.28^#^
RAD, mm	33.53 ± 3.67	46.42 ± 8.36*	39.59 ± 11.93
RVD, mm	19.30 ± 2.76	35.35 ± 8.65*	22.22 ± 5.03^#^
LVEF, %	69.27 ± 6.60	66.65 ± 7.07	68.22 ± 5.87

Values are expressed as mean ± standard deviation

*PH* pulmonary hypertension, *NYHA* New York Heart Association, *CPB* cardiopulmonary bypass, *LAD* left atrial diameter, *LVEDD* left ventricular end-diastolic diameter, *LVESD* left ventricular end-systolic diameter, *RAD* right atrial diameter, *RVD* right ventricle diameter, *LVEF* left ventricular ejection fraction

* *P* < 0.05 compared with control group

^#^ *P* < 0.05 compared with atrial septal defect group

### Blood sampling and flow cytometry

All patients preoperatively fasted overnight as did healthy volunteers. Blood samples were drawn and centrifuged to obtain platelet-poor plasma (PPP) [4]. 50 μL of PPP was incubated with 4 μl of anti-CD31-PE and 4 μL of anti-CD42b-FITC antibodies at room temperature for 20 min with gentle orbital shaking in the dark [4]. The samples incubated with corresponding isotype control (all from Beckman Coulter, France) were used as controls. After labeling, samples were analyzed via MoFlo XDP (Beckman coulter) by an independent examiner who was blinded to study arm and intentions. Before analysis, 50 μL flow count calibrator beads (Beckman Coulter) with known concentration provided by the manufacturer were added into the antibody-labeled tubes. After excluding non-specific fluorescence, those positively labeled by anti-CD31-PE and negatively labeled by anti-CD42b-FITC and <1 μm in size were considered to be EMPs [4].

### Generation of EMPs

EMPs were generated ex vivo by incubating human umbilical vein endothelial cells (HUVECs) with plasminogen activated inhibitor-1 (PAI-1) as previously described [2–6]. Briefly, passage 4 HUVECs were grown to confluence in T75 flasks coated with 1% gelatin in endothelial cell growth medium-2 (Clonetics) containing 20% fetal bovine serum. Cultured cells were maintained at 37 °C in 5% humidified CO_2_. After serum starvation, cells were stimulated with 10 ng/mL human PAI-1. Three hours later, the EMPs-rich supernatant was collected and centrifuged (300×*g*, 10 min) to remove cell debris. The supernatant was removed after ultracentrifugation (105×*g*, 60 min) and EMPs were resuspended in phosphate-buffered saline (PBS) at room temperature for subsequent experiments.

### SiRNA targeting P38 MAPK

To confirm the relationship between EMPs and the P38 MAPK pathway, we used targeted siRNAs to reduce expression of P38 MAPK in HUVECs. HUVECs were cultured in endothelial cell medium (ScienCell) supplemented with 5% Fetal bovine serum (FBS), 1% growth factors, and 1% penicillin/streptomycin. Cells were serum starved in 0.5% FBS over night before experiments. Targeted SiRNAs and scrambled siRNAs (negative control) (Dharmacon, MA) were transfected into cells with siPORT™ NeoFX™ Transfection Agent (Invitrogen, USA) and Opti-MEM I (Gibco). InitialsiRNA concentrations used were 70 nM. At 12 h after transfection, medium was exchanged with endothelial cell medium (Sciencell, Carlsbad, CA) consisting of 10% Fetal bovine serum and 10 ng/mL epidermal growth factor to remove siRNA. A separate control group containing only with siPORT™ NeoFX™ Transfection Agent and Opti-MEM I was also employed.

### ELISA for cell cultures

After siRNA transfection, HUVECs were cultured for an additional 48–72 h until the monolayer of cells reached 90–100% confluence. The cultured cells were then serum starved overnight and stimulated with EMPs (2 × 10^5^/mL) for 6 h. After centrifuging for 20 min at 12,000×*g*, the supernatants were collected and tumor necrosis factor-α (TNF-α) and interleukin (IL)-6 levels were evaluated using ELISA kits (ebioscience) as recommended in the manufacturer’s protocol.

### Animal experiments

All animal experiments were approved by the Animal Ethics Commission of the First Affiliated Hospital of Sun Yat-sen University. The investigation conformed to the provisions of the Declaration of Helsinki in 1995 (as revised in Edinburgh 2000). Eight-week-old female C57BL6 mice were obtained from the animal center of Sun Yat-sen University, north campus. 1 × 10^5^ or 5 × 10^5^/mL EMPs were injected into the mice via tail vein. Those injected with an equal volume of PBS were used as controls. 6 h after injection, mice were fully anesthetized with sodium pentobarbital (50 mg/kg) and blood samples were drawn for measurement of pro-inflammatory factors. In addition, the heart was isolated and frozen in liquid nitrogen for further immunoblotting analysis and immunohistochemical staining as previously described [3, 12, 13].

### ELISA for plasma

The blood samples drawn from mice were centrifuged and plasma was isolated. TNF-α and IL-6 concentrations were determined using ELISA kits (ebioscience).

### Immunoblotting analysis

To investigate the effects of EMPs on relevant proteins in the mouse heart, eNOS expression and phosphorylation at Ser1177, P38 MAPK expression and phosphorylation, and expression of caveolin-1 were assessed by immunoblotting with specific antibodies as previously described [3].

To investigate the effects of EMPs and siRNAs on cultured cells, HUVECs were evaluated without and with transfection of siRNAs targeting P38 MAPK (and scrambled control siRNAs) as described above. HUVECs were stimulated with EMPs (2 × 10^5^/mL) for 1 h. Then cellular proteins were harvested, total and phosphorylated P38 MAPK, and GAPDH protein expression were determined by immunoblotting as previously described [4, 14].

Antibodies for detection of phosphorylation of eNOS at Ser1177, P38 MAPK, phosphorylation of P38 MAPK, and caveolin-1 were purchased from Cell Signaling Technology (Danvers, MA). Anti-eNOS was obtained from Santa Cruz Biotechnology (Santa Cruz, CA). Anti-GAPDH was obtained from Proteintech Group (Chicago, IL). Proteins were separated by SDS-PAGE and then visualized using luminol reagent (Santa Cruz Biotechnology) according to standard methods.

### Immunohistochemical staining

To investigate the effects of EMPs on relevant protein expression in mouse hearts, isolated mouse hearts treated without or with EMPs were embedded with paraffin, sectioned and subjected to immunostaining. The expression of caveolin-1, eNOS, and P38 MAPK in mouse hearts was detected by immunohistochemical staining using standard protocols. Polycolonal rabbit IgG anti-Caveolin-1 antibody (1:100, Abcam), anti-eNOS antibody (1:100, Abcam) and anti-p38 MAPK antibody (1:100, Abcam) were used as primary antibodies.

### Measurement of nitric oxide (NO) generation

1 × 10^5^ or 5 × 10^5^/mL EMPs or equal volumes of PBS were injected into the mouse tail vein. 6 h later, hearts were isolated and placed in 1:9 (wt/vol) cold homogenization buffer. The hearts were cut into small pieces with an iris scissors and homogenized five times on ice (10 s with 30 s intervals between homogenizations). The homogenates were then centrifuged for 8 min (2000 rpm, 4 °C) and the supernatant was isolated. NO concentration was determined by measuring total nitrate plus nitrite (NO_3_^−^ + NO_2_^−^) using an NO detection kit (Nanjing Jiancheng Bioengineering Institute, Nanjing, China) according to the manufacturer’s instructions. Briefly, nitrate was enzymatically converted into nitrite by nitrate reductase, and nitrite was quantified with Griess reagent at an absorbance of 550 nm, as previously described [3]. The range of the detection of nitrate plus nitrite is 0–600 μmol/L according to the manufacturer. In addition, the total protein concentration of the supernatant was determined by bicinchoninic acid protein assay (Merck, Whitehouse Station, NJ).

### Statistical analysis

Statistical analyses were performed using Prism 5 software. For comparison of healthy subjects, ASD patients, and VSD patients, or the impact of different concentrations of EMPs on protein expression in hearts and cultured cells, one-way ANOVA and Newman-Keuls comparisons were used. Chi-square test was used to compare proportions between different groups. For comparison between EMPs and controls, a *t* test was used. P < 0.05 was considered to indicated statistical significance. Data were presented as mean ± SDEM.

## Results

### Demographic and clinical parameters

Demographic and clinical features are shown in Table 1. Age and gender were similar among the three groups of subjects. 15 patients with ASD and 6 patients with VSD were suffering from pulmonary hypertension. According to New York heart association classification, of the 20 patients with ASD 16 patients were class II and 4 patients were class III. Of 23 patients with VSD, 15 patients were class II and 8 patients were class III, respectively. The duration of CPB (66 ± 28 vs. 75 ± 23 min) and the time of aortic cross clamping (33 ± 24 vs. 39 ± 20 min) were similar among ASD group and VSD group during the operation, respectively.

### Echocardiographic findings

Transthoracic echocardiography differences found among the three subject groups are shown in Table 1. The control group had no evidence of morphologic heart disease. When compared with the control group, left atrial diameter (LAD), right atrial diameter (RAD) and right ventricular diameter (RVD) were significantly enlarged in ASD patients; left ventricular end-diastolic diameter (LVEDD), and left ventricular end-systolic diameter (LVESD) were significantly smaller in the ASD patients group. LAD and LVEDD were significantly enlarged in the VSD patients group. When compared with the ASD group, LVEDD and LVESD were significantly enlarged in the VSD group; RVD was significantly smaller in VSD group. There were no significant differences in left ventricular ejection fraction among the three groups of subjects.

### Levels of plasma EMPs

**Fig. 1:**
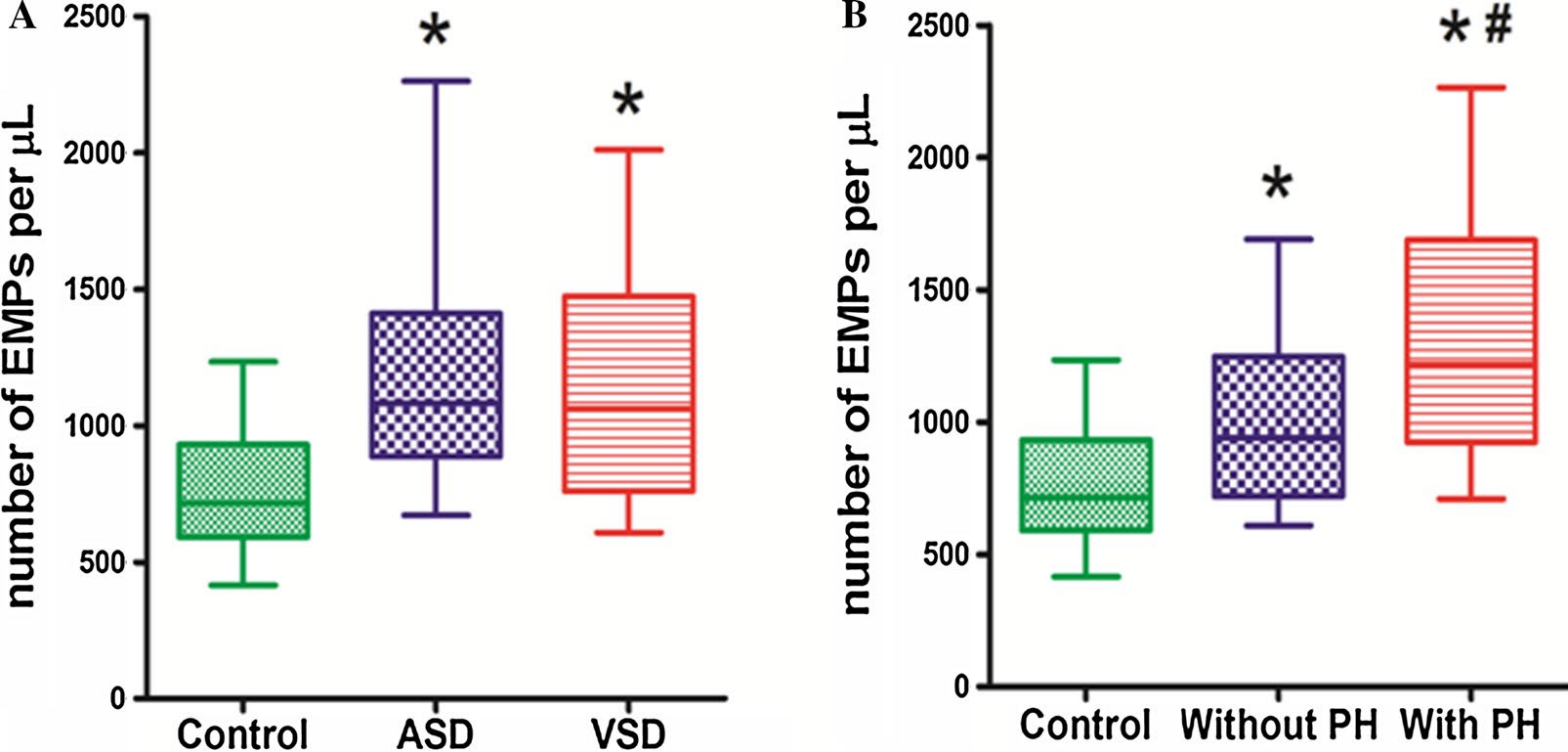
EMPs increase in patients with atrial septal defect and ventricular septal defect. **A** Compared with control group, EMPs were increased in patients with ASD and VSD. **B** EMPs were increased in patients with ASD and VSD associated with pulmonary hypertension (PH) than those without PH. *P < 0.05 compared with control group. ^#^ *P* < 0.05 compared with ASD and VSD patients

When compared with the control group, plasma EMPs levels were significantly elevated in the ASD group and VSD group (Fig. 1A). The higher levels of plasma EMPs in ASD and VSD patients were positively correlated with pulmonary hypertension (Fig. 1B).

### Effects of EMPs on expression of P38 MAPK, Caveolin-1 and eNOS in mouse hearts and in HUVECs

**Fig. 2:**
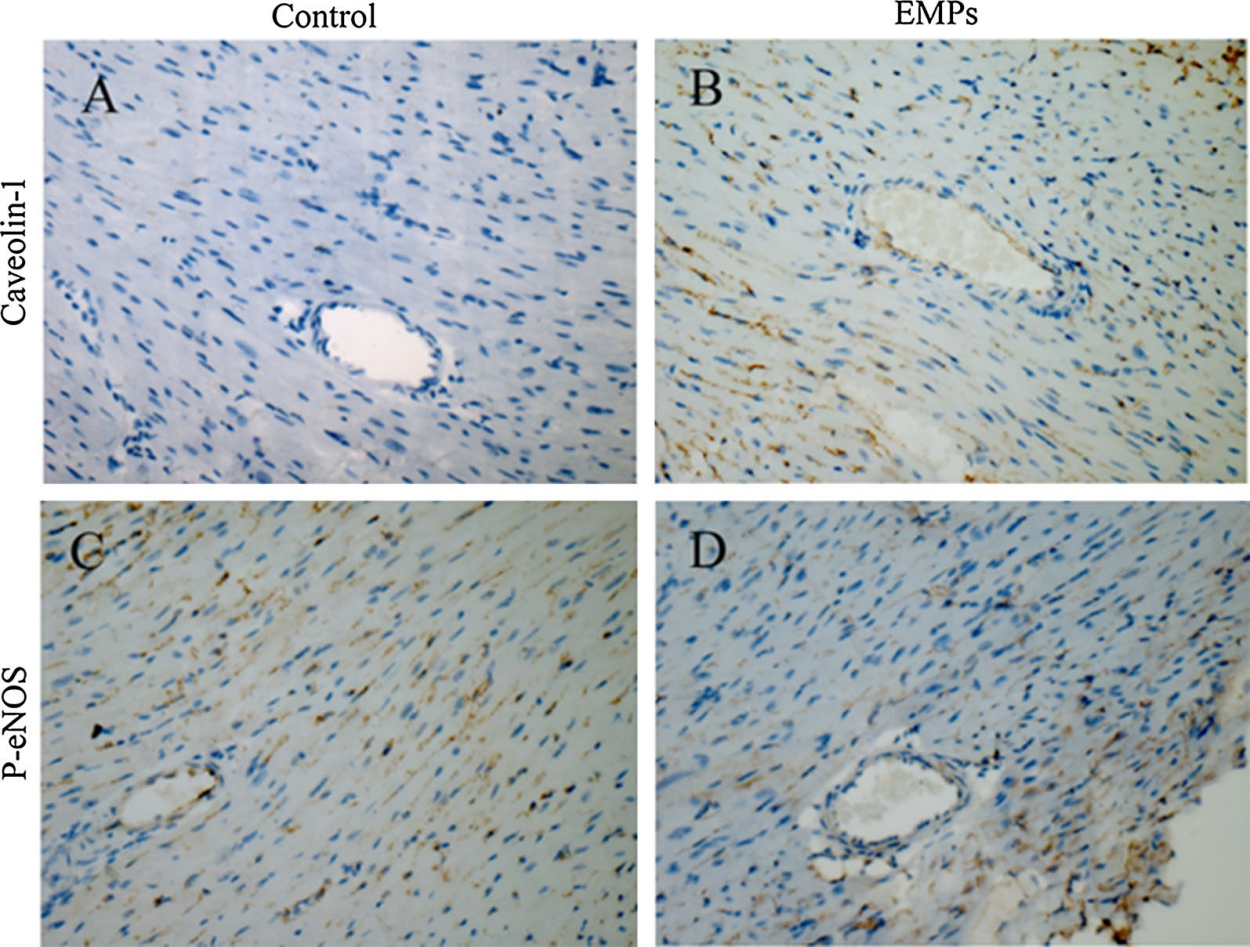
Effects of EMPs on Caveolin-1 and eNOS in the mouse hearts**. A**–**D** Immunohistochemical staining showed that EMPs can increase the expression of caveolin-1 and decrease the expression of phosphorylation of eNOS in the mouse hearts

**Fig. 3:**
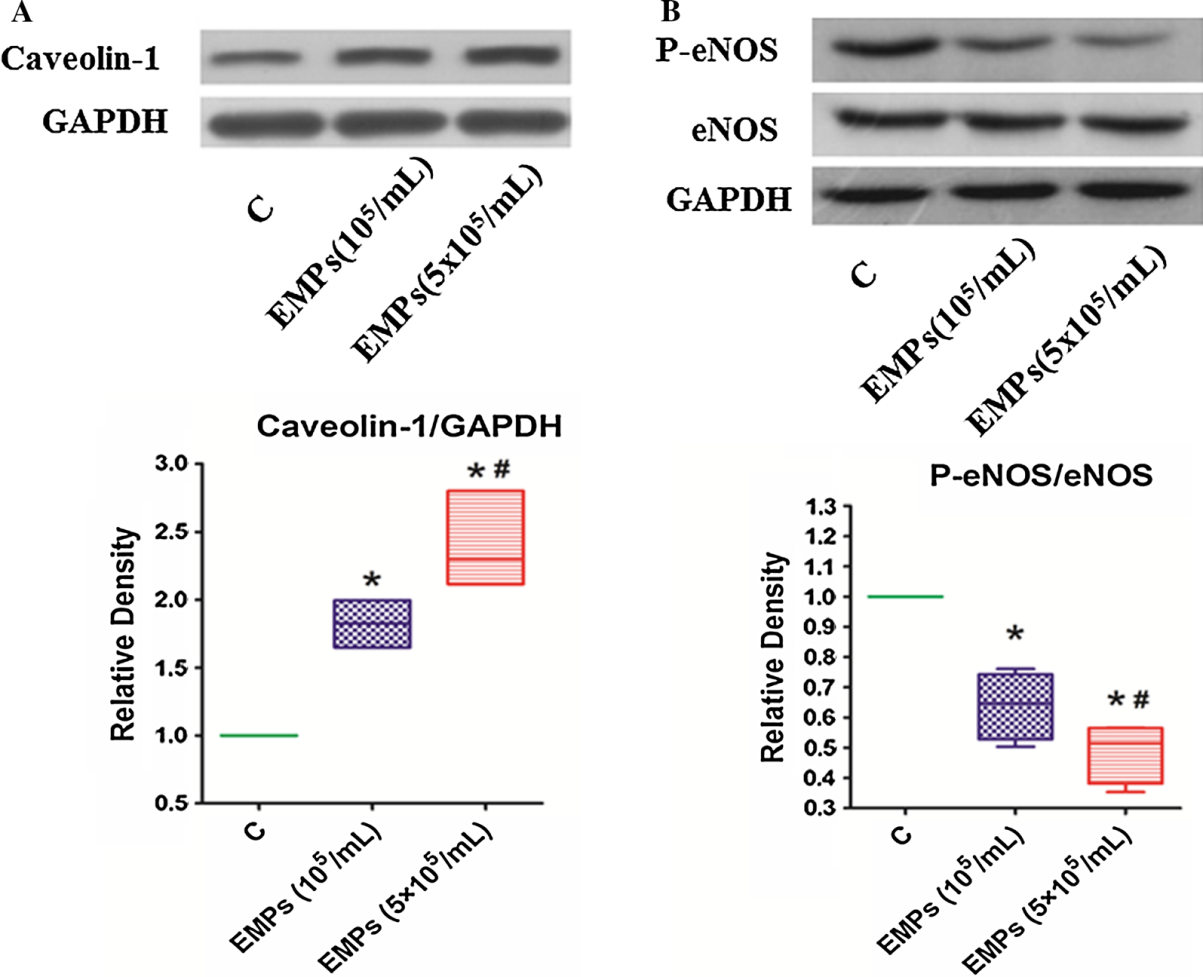
Effects of EMPs on Caveolin-1 and eNOS in the mouse hearts. **A**, **B** Immunoblotting showed that EMPs were significantly increased expression of caveolin-1 and decreased eNOS phosphorylation at S1177 site with a dose-dependent effect without altering the eNOS expression in the mouse hearts. (*P < 0.05 compared with control group. ^#^ *P* < 0.05 compared with group of EMPs 10^5^/mL, n = 8)

In mouse hearts, both immunohistochemical staining and immunoblotting showed that EMPs significantly increased expression of caveolin-1 (Figs. 2A, B; 3A). EMPs dramatically decreased eNOS phosphorylation at the S1177 site without altering total eNOS expression (Figs. 2C, D; 3B).

**Fig. 4:**
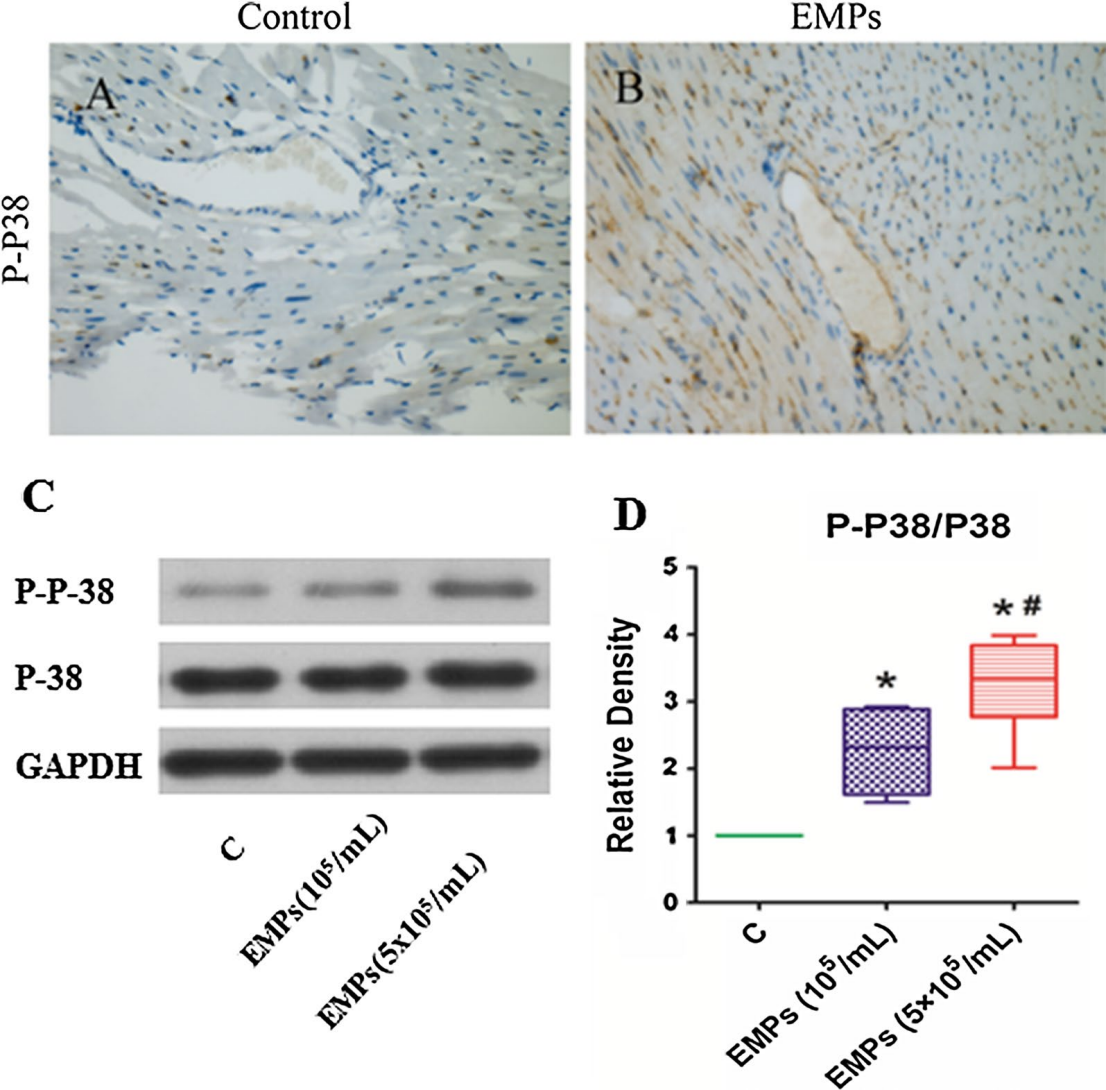
EMPs increased P38 phosphorylation in the mouse hearts. **A**, **B** Immunohistochemical staining showed that EMPs can increase the phosphorylation of P38 in the mouse hearts. **C**, **D** Immunoblotting showed that EMPs were dramatically increased P38 phosphorylation with a dose-dependent effect. (*P < 0.05 compared with control group, ^#^ *P* < 0.05 compared with the group of EMPs 10^5^/mL, n = 8)

**Fig. 5:**
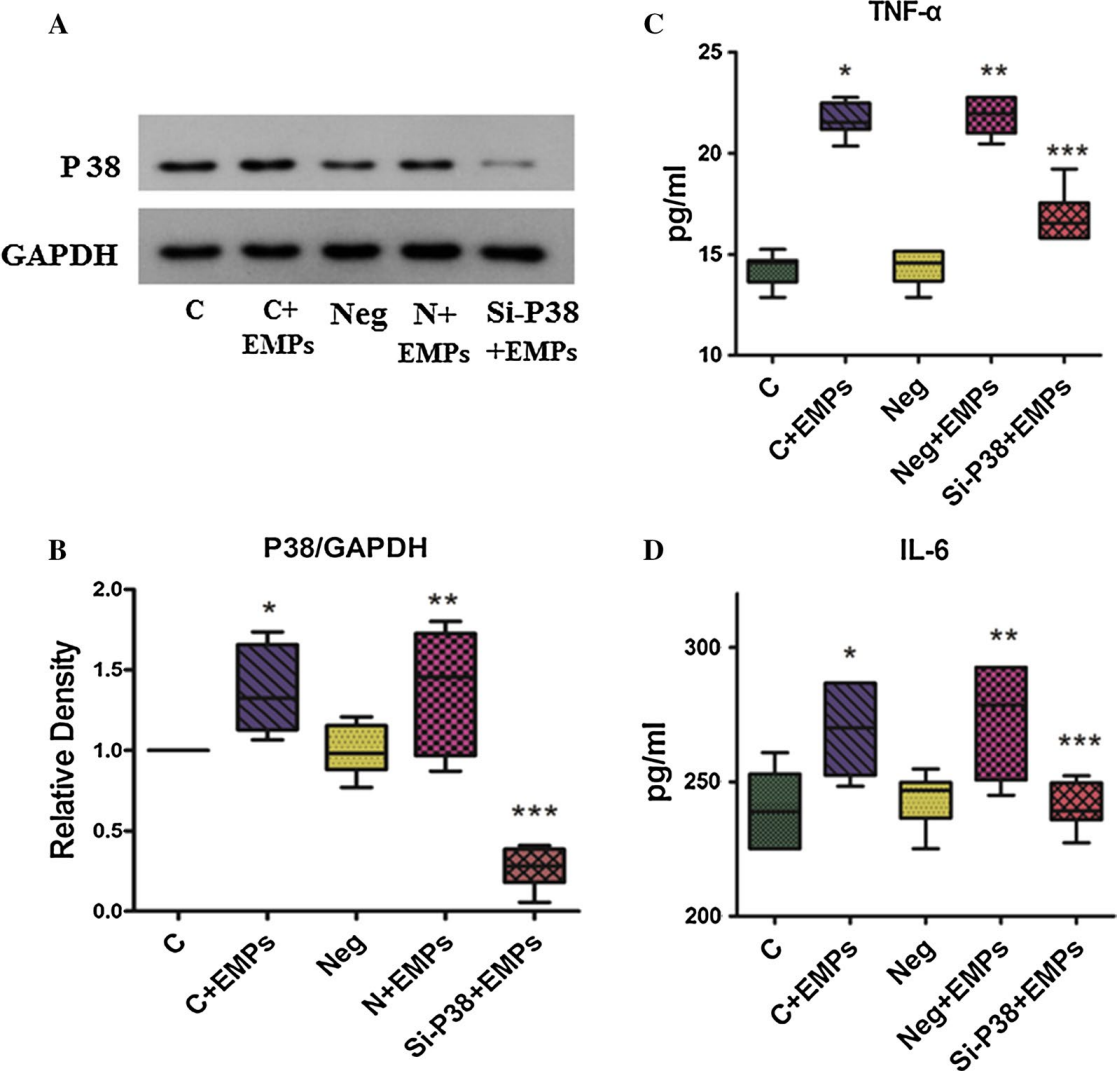
EMPs can stimulate TNF-α and IL-6 release in HUVECs, which can be down-regulated by P38-siRNAs. **A**, **B** Immunoblotting showed EMPs can increase P38 expression, which can be reduced by siRNAs targeting P38 MAPK (Si-P38). **C**, **D** TNF-α and IL-6 concentration were relative low in control (**C**) and negative (Neg) group. After 6 h stimulated with EMPs, TNF-α and IL-6 level increased in each group. TNF-α and IL-6 level in siRNAs P38 MAPK group was significantly lower than that in non-P38 siRNAs interfering groups. (*P < 0.05 compared with control group, **P < 0.05 compared with negative group, ***P < 0.05 compared with the group of EMPs 10^5^/mL, n = 8)

Both immunohistochemical staining and immunoblotting showed that EMPs dramatically increased P38 MAPK phosphorylation (Fig. 4). EMPs also stimulated increased expression of P38 MAPK in HUVECs. This was substantially inhibited by siRNAs targeting P38 MAPK (Fig. 5A, B).

### Effects of EMPs on TNF-α and IL-6 concentrations in cultured HUVECs

EMPs significantly stimulated an increase in TNF-α concentration in cultured HUVECs. In EMPs stimulated groups, TNF-α concentration in the group transfected with siRNA targeting P38 MAPK was significantly lower than in cells transfected with the scrambled siRNA control. No differences were observed between control siRNA and cells not treated with siRNA (Fig. 5C). A similar pattern was observed with concentrations of IL-6 (Fig 5D).

### Effects of EMPs on TNF-α and IL-6 concentrations in mouse plasma

**Fig. 6:**
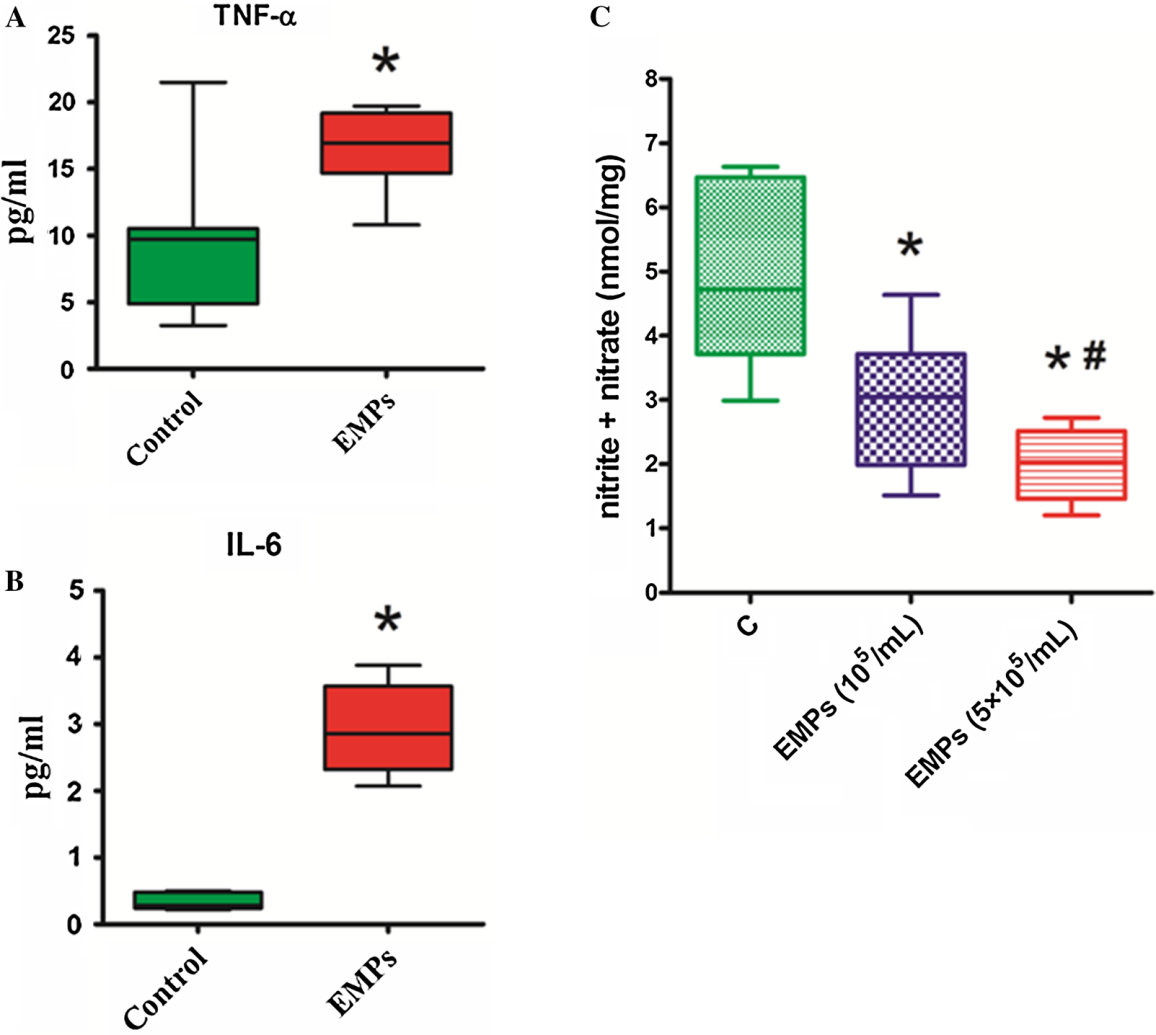
EMPs increased TNF-α and IL-6 release in mouse and decreased NO generation in the mouse hearts. **A**, **B** The levels of TNF-α and IL-6 in mouse plasma in EMPs group (5 × 10^5^/mL) were significantly higher than that in control groups. (*P < 0.05 compared with control group, n = 8). **C** EMPs significantly decrease NO production in the mouse hearts. The group of EMPs (5 × 10^5^/mL) was released NO less than the group of EMPs (10^5^/mL). (*P < 0.05 compared with control group, ^#^ *P* < 0.05 compared with group of EMPs (10^5^/mL), n = 12)

As shown in Fig. 6A and B, TNF-α and IL-6 concentrations in the plasma of mice were significantly increased after intravenous injection with EMPs 5 × 10^5^/mL for 6 h (when compared with controls).

### Effects of EMPs on NO generation in mouse hearts

As shown in Fig. 6C, NO concentration in the mouse hearts treated with EMPs was significantly decreased when compared with controls. There was a dose-dependent effect of EMPs to stimulate NO production in mouse hearts (Fig. 6C).

## Discussion

We and others have previously demonstrated that EMPs levels are elevated in patients with valvular heart diseases and this contributes to impairing mitral valve endothelial cell function [4]. We further showed that circulating microparticles generated from patients with valvular heart disease and cardiac surgery can contribute to endothelial dysfunction [1]. In the present study, we are first to show that EMPs levels are also increased in adult patients with ASD and VSD resulting in increased inflammation and endothelial dysfunction. This indicates that EMPs are not only increased in acquired heart diseases, but also elevated in simple acyanotic congenital heart diseases where it may play a pathophysiological role.

The mechanisms underlying increased numbers of plasma EMPs in ASD and VSD are unclear. Previous studies demonstrated that EMPs can be released from endothelial cell activation and/or apoptosis [15]. The atrium and ventricle sizes were increased in ASD and VSD patients. These changes and additional septal defects lead to hemodynamics change and shear stress that may potentially contribute to EMP formation [15, 16]. Shear stress is a major determinant of endothelial damage which may induce activation of endothelial cells and lead to EMPs release [16]. It is also possible that abnormal hemodynamic forces including abnormal blood flow through cardiac defects and increased blood flow in cardiopulmonary circulation may stimulate EMPs release [15].

Pulmonary hypertension is one major complications of congenital heart diseases [17, 18]. In the present study, we found that levels of EMPs were higher in patients with ASD and VSD accompanied by pulmonary hypertension than in those without pulmonary hypertension. Endothelial cell activation and apoptosis is observed in patients with pulmonary hypertension [19, 20] leading to increases in pulmonary blood flow that exert abnormal shear stress and result in endothelial dysfunction in lamb models [21]. These abnormalities may cause more EMPs release in ASD and VSD patients with pulmonary hypertension than in isolated ASD and VSD patients without pulmonary hypertension. Samadja reported no significant differences in EMPs level between irreversible and reversible pulmonary arterial hypertension in children with congenital heart diseases. Thus, it is possible that EMPs levels increase when pulmonary hypertension occurs but does not further increase upon progression to irreversible pulmonary arterial hypertension. This may be due to the fact that endothelial cells are already activated to release EMPs at a maximal rate in reversible pulmonary hypertension and further increases to generate more EMPs do not occur as disease progresses to the irreversible state. Taken together, this data suggests that increased level of EMPs may be a predictor for pulmonary hypertension in congenital heart disease [22]. Indeed, EMPs are already considered as marker of many other diseases. Our findings extend these previous results to suggest that EMPs may also be a biomarker of pulmonary hypertension in adult congenital heart diseases and congenital heart diseases per se.

We and others have been demonstrated that EMPs may play an important role in exacerbating endothelial dysfunction [2–4, 7]. It is well known that eNOS-mediated NO production plays a critical role in the regulation of endothelial function [23, 24]. To investigate the mechanisms by which EMPs impair endothelial function, we performed some basic studies in mice and endothelial cells. We found that EMPs significantly inhibited eNOS phosphorylation and NO production in mouse hearts in a dose-dependent manner. Caveolin-1 binds to eNOS to keep eNOS in an inactive state [23]. We found that caveolin-1 expression in mouse hearts was increased by EMPs in a dose-dependent manner. This suggests that EMPs may inhibit eNOS activity to block NO generation in mice hearts through an increase in caveolin-1 expression. As reduction of NO bioactivity is a hallmark of endothelial dysfunction, our findings indicate that EMPs may inhibit eNOS activity to reduce NO to contribute to endothelial dysfunction in hearts. It should be noted that iNOS is another major source of NO. EMPs may also inhibit iNOS to produce NO. However, we found that EMPs didn’t inhibit iNOS expression in the healthy and hypercholesterolemic mice hearts in both the current study and previous study (data not shown) [3]. This suggests that iNOS is probably not a major contributor to NO reduction observed in our studies. We recently demonstrated that circulating microparticles from valvular heart diseases and cardiac surgery can inhibit NO generation to impair endothelium dependent vasodilation [1]. As EMPs are part of circulating microparticles, our findings that plasma EMPs level increased and EMPs inhibit NO generation in the hearts suggest that EMPs may affect hemodynamic stability in patients with ASD and VSD post cardiac surgery. In addition, as EMPs inhibit NO production, this may also exacerbate the development of pulmonary hypertension leading to a further increase in EMPs.

Perioperative systemic inflammation is one of the major risk factors and complications in cardiac surgery. P38 MAPK signaling mediates myocardial inflammatory responses and endothelial dysfunction that can contribute to myocardial damage following acute injury [25, 26]. P38 MAPK signaling pathway also plays a vital role in the formation and maturation process of EMPs [27]. In the present study, our data showed that phosphorylation of p38 MAPK in mouse hearts was increased by EMPs in a dose-dependent manner. This suggests that EMPs can activate the P38 MAPK pathway. We further found that EMPs also increased P38 MAPK expression and stimulated increases in TNF-α and IL-6 levels both in cultured endothelial cells and mice. The increased P38 MAPK expression as well as downstream TNF-α and IL-6 production was inhibited by disrupting the p38 MAPK pathway with targeted specific siRNAs. This suggests that EMPs stimulate TNF-α and IL-6 production using a P38 MAPK-dependent pathway consistent with other studies of pro-inflammatory signaling related to TNF-α and IL-6 production [26, 28]. In some studies, EMPs level is correlated with serum IL-6 level, in both healthy subjects and in patients with coronary heart disease [29, 30]. In vitro studies show that inflammatory factors induce endothelial cell apoptosis leading to production of EMPs. Our study showed that EMPs can induce endothelial cells to release pro-inflammatory factors in a P38 MAPK-dependent fashion. Thus, EMPs may be both the cause and consequence of pro-inflammatory responses in a feed-forward mechanism that amplifies inflammation and its pathophysiological consequences. This raises the possibility that blocking EMPs’ function or reducing EMPs’ level may be a potential therapeutic strategy for controlling inflammation in congenital heart disease. Since TNF-α and IL-6 are secretory mediators and proinflammatory cytokines that increase endothelial cell permeability and promote endothelial cell activation, dysfunction, and apoptosis [25, 26], our data suggests that EMPs may induce inflammation to impair endothelial function and damage vascular endothelial cells by activating the P38 MAPK pathway to promote TNF-α and IL-6 production. Thus, future studies will be investigated whether EMPs upregulate some gene expressions to activate P38 MAPK pathway in endothelial cells.

Study Limitations: Due to the limitations in current technology, we are unable to extract EMPs from patients’ plasma directly to perform animal or cell studies. Instead, in the present study, we have relied on a published strategy to obtain EMPs from cultured endothelial cells stimulated with PAI-1 [2, 4, 6] that may cause xenotransplant-type reactions. In addition, HUVECs used in the present study may not be representative of all human endothelial cell types and components of EMPs from different endothelial cell sources may have different functional abilities with diverse biological functions [5, 6]. Thus, EMPs used in the current study may have some differences from EMPs in congenital heart disease patients. Furthermore, the mice used in the current study were healthy mice and its physiology may not represent human congenital heart disease (patho) physiology. However, the primary goal of the present study was to determine effects of EMPs on mouse heart, independent of pathology. If EMPs can adversely affect healthy hearts, it seems plausible that effects of EMPs in congenital heart disease may be even more severe. Our data clearly demonstrated EMPs impaired heart function. We only tested effects of EMPs on endothelial cells because we wanted to focus on endothelial function, a major line of investigation in our lab. This represents an additional study limitation. Whether EMPs have similar effects on human cardiomyocytes needs to be evaluated in subsequent studies beyond the scope of the present study. Based on our previous findings and other publications [1–6], we speculate that EMPs may induce inflammation leading to endothelial dysfunction in patients with congenital heart diseases. Identifying and evaluating safe P38 MAPK inhibitors to block P38 MAPK-dependent pathways in vivo as a potential therapeutic strategy to decrease EMPs are beyond the scope of the present study but are the subject of future studies in our lab. Other future related studies include evaluating relationships between EMPs level and cardiopulmonary bypass, deep hypothermic circulatory arrest, and postoperative complications.

## Conclusions

In summary, our study demonstrated that EMPs were increased in adult patients with congenital heart diseases. These increased EMPs inhibited eNOS activity to reduce NO production and activated P38 MAPK-dependent signaling pathways to promote production of TNF-α and IL-6. These mediators of pro-inflammatory response impaired endothelial function. These changes may contribute to impairment of myocardial and vascular function during the perioperative period to affect hemodynamic stability after cardiac surgery. In addition, the consequences of increased EMPs may also promote development of pulmonary hypertension. We conclude that the P38 MAPK pathway may play a role in EMPs-induced inflammation and provide a potential therapeutic target in surgery of congenial heart disease.

## Additional file

**Additional file 1.** Online supplemental materials and methods.

## Abbreviations

EMPs: endothelial microparticles
ASD: atrial septal defect
VSD: ventricular septal defect
HUVECs: human umbilical vascular endothelial cells
eNOS: endothelial nitric oxide synthase
TNF-α: tumor necrosis factor-α
IL-1: interleukin-1
CPB: cardiopulmonary bypass
NO: nitric oxide
NYHA: New York Heart Association
LAD: left atrial diameter
LVEDD: left ventricular end-diastolic diameter
LVESD: left ventricular end-systolic diameter
RAD: right atrial diameter
RVD: right ventricle diameter
LVEF: left ventricular ejection fraction
