# Supplementary material for: Endothelial microparticles are increased in congenital heart diseases and contribute to endothelial dysfunction
Source: J Transl Med. 2017 Jan 4;15:4. doi: 10.1186/s12967-016-1087-2 (PMC5210308; doi:10.1186/s12967-016-1087-2)
Supplement: Supplementary file 1 — Additional file 1. Online supplemental materials and methods. [file 12967_2016_1087_MOESM1_ESM.doc]

**Endothelial microparticles increased in congenital heart diseases and impaired endothelial function**

Ze-Bang Lin, MD^1,4,5^*, Hong-Bo Ci, MD, PhD ^1,4,5^*, Yan Li, PhD^1,4,5^, Tian-Pu Cheng, BS^1,4,5^, Dong-Hong Liu, MD,PhD^2^, Yan-Sheng Wang, MD,PhD^7-9^, Jun Xu, MD,PhD^7-9^, Hao-Xiang Yuan, MD^1,4,5^, Hua-Ming Li, MD^1,4,5^, Jing Chen, MD^3-^, Li Zhou, MD^1,4^, Zhi-Ping Wang, MD, PhD^1,4^, Xi Zhang,MD^1,4^, Zhi-Jun Ou, MD^3-5#^, Jing-Song Ou, MD, PhD^1,4-6#^.

Division of Cardiac Surgery, ^1^

The First Affiliated Hospital of Sun Yat-sen University,

Department of Ultrasound,^2^

The First Affiliated Hospital of Sun Yat-sen University,

Division of Hypertension and Vascular Diseases,^3^

The First Affiliated Hospital of Sun Yat-sen University,

The key Laboratory of Assisted Circulation, Ministry of Health,^4^

National and Guangdong Province Joint Engineering Laboratory for Diagnosis and Treatment of Vascular Diseases, ^5^

Guangdong Provincial Key Laboratory of Brain Function and Disease^6^

Guangzhou, 510080, P.R. China

State Key Laboratory of Respiratory Disease, ^7^

Guangzhou Institute of Respiratory Disease,^8^

The First Affiliated Hospital of Guangzhou Medical University^9^

Guangzhou, 510120, P.R. China

* These two authors contributed equally to this study.

# Both Zhi-Jun Ou and Jing-Song Ou contributed equally to the design and supervision of the research for this article.

Address correspondence and reprint requests to

Jing-Song Ou, MD, Ph.D

Division of Cardiac Surgery

The First Affiliated Hospital, Sun Yat-sen University

58 Zhong Shan Er Road,

Guangzhou, 510080,

P.R. China

Tel: 86-20-87755766-8238

Fax: 86-20-87333122

Email: [oujs@mail.sysu.edu.cn](mailto:oujs@mail.sysu.edu.cn), [oujs2000@yahoo.com](mailto:oujs2000@yahoo.com).

**Online supplemental materials and methods**

**Study population**

20 patients diagnosed with ASD, 23 patients with VSD and 30 age-matched healthy volunteers were recruited at The First Affiliated Hospital of Sun Yat-sen University. Patients with diseases which may increase EMPs level, including coronary heart disease, hypertension, infectious disease, severe trauma, antibiotic therapy, lupus anticoagulant, multiple sclerosis, renal failure, rheumatic diseases in acute stage and valvular heart disease were excluded. Healthy volunteers below 18 years old and those abused alcohol and/or heavy smokers were excluded. This study was approved by Ethics Committee of The First Affiliated Hospital, Sun Yat-sen University. Informed consents were obtained from all subjects enrolled in this study. Clinical characteristic, doppler echocardiographic variables (the definition and classification of pulmonary hypertension are referenced as 2014 Nice Pulmonary Hypertension Classification System) and operation data were collected and described in Table 1.

**Blood samplings and flow cytometry**

All patients preoperatively and healthy volunteers fasted overnight. Blood samples were drawn and centrifuged to gain platelet-poor plasma (PPP). 50μl of PPP was incubated with 4μl of anti-CD31-PE and 4μl of anti-CD42b-FITC antibodies at room temperature for 20 min with gentle orbital shaking in the dark. The samples incubated with corresponding isotype control (all from Beckman coulter, France) were used as controls. After labeling, samples were analyzed via MoFlo XDP (Beckman coulter) by an independent examiner with no knowledge of the intentions for this study. Before analyzation, 50μl flow count calibrator beads (Beckman Coulter) with known concentration provided by manufacturer were added into the antibody-labeled tubes. After excluding non-specific fluorescence, those positively labeled by anti-CD31-PE and negatively labeled by anti-CD42b-FITC and <1μm in size were defined as EMPs.

**Generation of EMPs**

EMPs were generated by incubating human umbilical vein endothelial cells (HUVECs) with plasminogen activated inhibitor-1 (PAI-1) as previously described. Briefly, passage 4 HUVECs were grown to confluence in T75 flasks coated with 1% gelatin in endothelial cell growth medium-2 (Clonetics) containing 20% fetal bovine serum. Cultured cells were maintained at 37°C in 5% humidified CO_2_. After serum starvation, cells were stimulated with 10 ng/mL human PAI-1. Three hours later, the EMPs-rich supernatant was collected and centrifuged (300*g*, 10 min) to remove cell debris. The supernatant was removed after ultracentrifugation (10^5^*g*, 60 min) and EMPs were resuspended in phosphate-buffered saline (PBS) at room temperature for subsequent experimentation**s**.

**SiRNA interfering for P38 MAPK**

HUVECs were cultured in endothelial cell medium (ScienCell) supplemented with 5% Fetal bovine serum (FBS), 1% growth factors, and 1% penicillin/streptomycin. Cells were starved with 0.5% FBS over night before experiments. To confirm the relationship of EMPs and P38/MAPK pathway, we knocked down the MAPK14 in HUVECs by specific small interfering RNAs (siRNAs). SiRNAs and negative control siRNA (purchased from Dharmacon) were transfected with siPORT™ NeoFX™ Transfection Agent (Invitrogen, USA) and Opti-MEM I (Gibco) as recommended in the instruction protocol. The siRNA concentrations were 70nM. At 12 hours after transfection, medium was changed for endothelial cell medium (ECM, Sciencell, Carlsbad, CA) consist of 10% FBS and 10ng/ml EGF to remove siRNA. Control group contains only with siPORT™ NeoFX™ Transfection Agent and Opti-MEM I. Negative group contains with siPORT™ NeoFX™ Transfection Agent and Opti-MEM I, as well as negative control siRNA.

**ELISA**

After siRNAs transfection, HUVECs were cultured for additional 48 to 72 hours when the monolayer of cells reached at 90% to 100% confluence. The cultured cells were then serum starved overnight and stimulated with EMPs (2×10^5^/ml) for 6 hours. After centrifuging for 20 min at 12,000g, the supernatants were collected and tumor necrosis factor-α (TNF-α) and interleukin (IL)-6 were tested using ELISA kits (ebioscience) as recommended in the protocol.

**Animal experiments**

All animal experiments were approved by the Animal Ethnic Commission of the First Affiliated Hospital of Sun Yat-sen University. The investigation conformed to the provisions of the Declaration of Helsinki in 1995 (as revised in Edinburgh 2000). Eight-week-old female C57BL6 mice were obtained from the animal center of Sun Yat-sen University, north campus. 1×10^5^/mL or 5×10^5^/mL EMPs were injected to the mice via the tail vein. Those injected with equal amount of PBS were used as controls. 6 hours later, mice were fully anesthetized with sodium pentobarbital (50 mg/kg) and the blood samples were drawn for inflammatory factors measurement, and the heart was isolated and frozen in liquid nitrogen for further western blot analysis and immunohistochemical staining as previously described**.**

**ELISA for plasma**

The blood samples drawn from mice were centrifuged and plasma was isolated. TNF-α and IL-6 were determined using ELISA kits (ebioscience) as recommended in the protocol as mentioned above.

**Western blot analysis**

To investigate the proteins’ effects of EMPs on the mice heart, eNOS expression and phosphorylation at Ser1177, P38 expression and phosphorylation and expression of caveolin-1were assessed by western blot as described previously. Briefly, frozen heart samples were pulverized and placed in a modified RIPA buffer. The mixture was then homogenized and sonicated to break the cells, and the cell debris was removed by centrifugation at 14,000 *g* for 10 min at 4°C. The supernatant was transferred to a cold microcentrifuge tube, and protein concentrations were determined by a bicinchoninic acid protein assay. The protein was used for Western blot analysis.

To investigate the effects of EMPs and siRNAs on the cultured cells, cultured HUVECs and P38 siRNAs were manipulated as mentioned above. When the monolayer of interfered HUVECs reached 90% to 100% confluence, serum starved cells were stimulated with EMPs (2×10^5^/ml) for 1 hour. Then cellular proteins were harvested, total P38, phosphorylation of P38 and GAPDH protein levels were determined by western blot as described previously.

Antibodies for detection of phosphorylation of eNOS at Ser1177, P38, phosphorylation of P38 and caveolin-1 were purchased from Cell Signaling Technology (Danvers, MA). Anti-eNOS was bought from Santa Cruz Biotechnology (Santa Cruz, CA). Anti-GAPDH was bought from Proteintech Group (Chicago, IL). Proteins were visualized using a western blotting luminol reagent (Santa Cruz Biotechnology).

**Immunohistochemical Staining**

To investigate the proteins’ effects of EMPs in the mice hearts, the isolated mice heart treated with/without EMPs were embedded with paraffin and sectioned. The expression of caveolin-1, eNOS and P38 in the mice hearts were detected by immunohistochemical staining. Polycolonal rabbit IgG anti-Caveolin-1 antibody (1:100, Abcam), anti-eNOS antibody (1:100, Abcam) and anti-p38 antibody (1:100, Abcam) were used.

**Measurement of nitric oxide (NO) generation**

1× 10^5^/mL or 5×10^5^/mL EMPs or equal amount of PBS were injected to the mice via the tail vein. 6 hours later, the hearts were isolated and placed in 1:9 (wt/vol) cold homogenization buffer. The hearts were cut into small pieces with an iris scissors and homogenized five times on ice (10 s with 30 s intervals between homogenizations). The homogenates were then centrifuged at for 8 minutes (2,000 rpm, 4°C) and the supernatant was gained. NO concentration was determined by measuring total nitrate plus nitrite (NO3-+ NO2-) with an NO detection kit (Nanjing Jiancheng Bioengineering Institute, Nanjing, China) according to the manufacturer’s instructions. Briefly, nitrate was enzymatically converted into nitrite by nitrate reductase, and nitrite was quantified with Griess reagent at an absorbance of 550 nm, as previously described. The range of the detection of nitrate plus nitrite is 0-600 μmol/L according to the manufacturer. Besides, the protein concentration of the supernatant was determined by bicinchoninic acid protein assay (Merck, Whitehouse Station, NJ).

**Statistical analysis**

Statistical analyses were performed using Prism 5 software. For comparison of healthy subjects, ASD patients and VSD patients, or the impact of EMPs with different concentration on the heart and cultured cells, one-way ANOVA and Newman-Keuls were used. Chi-square test was used to compare proportions between different groups. For only comparison between EMPs and control, t test was used. p < 0.05 was considered statistically significant. Data were presented as mean ± SD

**Reference**

1. Fu L, Hu XX，Lin ZB，et al. Circulating microparticles from patients with valvular heart disease and cardiac surgery inhibit endothelium-dependent vasodilation. J Thorac Cardiovasc Surg. 2015;150:666-672.
2. Densmore JC, Signorino PR, Ou J, et al. Endothelium-derived microparticles induce endothelial dysfunction and acute lung injury. Shock. 2006;26:464-471.
3. Ou ZJ, Chang FJ, Luo D, et al. Endothelium-derived microparticles inhibit angiogenesis in the heart and enhance the inhibitory effects of hypercholesterolemia on angiogenesis. Am J Physiol Endocrinol Metab. 2011;300:E661-668.
4. Ci HB, Ou ZJ, Chang FJ, et al. Endothelial microparticles increase in mitral valve disease and impair mitral valve endothelial function. Am J Physiol Endocrinol Metab. 2013;304:E695-702.
5. Amabile N, Heiss C, Real WM, et al. Circulating endothelial microparticle levels predict hemodynamic severity of pulmonary hypertension. American journal of respiratory and critical care medicine. 2008;177:1268-1275.
6. Smadja DM, Gaussem P, Mauge L, et al. Comparison of endothelial biomarkers according to reversibility of pulmonary hypertension secondary to congenital heart disease. Pediatric cardiology. 2010;31:657-662.
7. He GW. Endothelial function related to vascular tone in cardiac surgery. Heart Lung Circ. 2005;14:13-18.
8. Floh AA, Manlhiot C, Redington AN, et al. Insulin resistance and inflammation are a cause of hyperglycemia after pediatric cardiopulmonary bypass surgery. J Thorac Cardiovasc Surg. 2015;150:498-504 e491.
9. Preventza O, Garcia A, Cooley DA, et al. Reoperations on the total aortic arch in 119 patients: short- and mid-term outcomes, focusing on composite adverse outcomes and survival analysis. J Thorac Cardiovasc Surg. 2014;148:2967-2972.
10. Peterson DB, Sander T, Kaul S, et al. Comparative proteomic analysis of PAI-1 and TNF-alpha-derived endothelial microparticles. Proteomics. 2008;8:2430-2446.
11. Sander TL, Ou JS, Densmore JC, et al. Protein composition of plasminogen activator inhibitor type 1-derived endothelial microparticles. Shock. 2008;29:504-511.
12. Barst RJ, Ertel SI, Beghetti M, Ivy DD. Pulmonary arterial hypertension: a comparison between children and adults. Eur Respir J. 2011;37:665-677.
13. Almansob MA, Xu B, Zhou L, et al. Simvastatin reduces myocardial injury undergoing noncoronary artery cardiac surgery: a randomized controlled trial. Arteriosclerosis, thrombosis, and vascular biology. 2012;32:2304-2313.
14. Chang FJ, Yuan HY, Hu XX, et al. High density lipoprotein from patients with valvular heart disease uncouples endothelial nitric oxide synthase. J Mol Cell Cardiol. 2014;74:209-219.
15. Takeshita J, Mohler ER, Krishnamoorthy P, et al. Endothelial cell-, platelet-, and monocyte/macrophage-derived microparticles are elevated in psoriasis beyond cardiometabolic risk factors. J Am Heart Assoc. 2014;3:e000507.
16. Jenkins NT, Padilla J, Boyle LJ, Credeur DP, Laughlin MH, Fadel PJ. Disturbed blood flow acutely induces activation and apoptosis of the human vascular endothelium. Hypertension. 2013;61:615-621.
17. Vion AC, Ramkhelawon B, Loyer X, et al. Shear stress regulates endothelial microparticle release. Circ Res. 2013;112:1323-1333.
18. Opotowsky AR. Clinical evaluation and management of pulmonary hypertension in the adult with congenital heart disease. Circulation. 2015;131:200-210.
19. Latus H, Yerebakan C, Schranz D, Akintuerk H. Right ventricular failure from severe pulmonary hypertension after surgery for shone complex: back to fetal physiology with reducting, atrioseptectomy, and bilateral pulmonary arterial banding. J Thorac Cardiovasc Surg. 2014;148:e226-228.
20. White K, Dempsie Y, Caruso P, et al. Endothelial apoptosis in pulmonary hypertension is controlled by a microRNA/programmed cell death 4/caspase-3 axis. Hypertension. 2014;64:185-194.
21. Tian W, Jiang X, Tamosiuniene R, et al. Blocking macrophage leukotriene b4 prevents endothelial injury and reverses pulmonary hypertension. Sci Transl Med. 2013;5:200ra117.
22. Tian J, Fratz S, Hou Y, et al. Delineating the angiogenic gene expression profile before pulmonary vascular remodeling in a lamb model of congenital heart disease. Physiological genomics. 2011;43:87-98.
23. Diehl P, Aleker M, Helbing T, et al. Increased platelet, leukocyte and endothelial microparticles predict enhanced coagulation and vascular inflammation in pulmonary hypertension. Journal of thrombosis and thrombolysis. 2011;31:173-179.
24. Chen Z, Bakhshi FR, Shajahan AN, et al. Nitric oxide-dependent Src activation and resultant caveolin-1 phosphorylation promote eNOS/caveolin-1 binding and eNOS inhibition. Molecular biology of the cell. 2012;23:1388-1398.
25. Xuan C, Chang FJ, Liu XC, et al. Endothelial nitric oxide synthase enhancer for protection of endothelial function from asymmetric dimethylarginine-induced injury in human internal thoracic artery. J Thorac Cardiovasc Surg. 2012;144:697-703.
26. Sarov-Blat L, Morgan JM, Fernandez P, et al. Inhibition of p38 mitogen-activated protein kinase reduces inflammation after coronary vascular injury in humans. Arteriosclerosis, thrombosis, and vascular biology. 2010;30:2256-2263.
27. Liang Y, Li X, Zhang X, et al. Elevated levels of plasma TNF-alpha are associated with microvascular endothelial dysfunction in patients with sepsis through activating the NF-kappaB and p38 mitogen-activated protein kinase in endothelial cells. Shock. 2014;41:275-281.
28. Curtis AM, Wilkinson PF, Gui M, Gales TL, Hu E, Edelberg JM. p38 mitogen-activated protein kinase targets the production of proinflammatory endothelial microparticles. Journal of thrombosis and haemostasis : JTH. 2009;7:701-709.
29. Yang S, Zhong Q, Qiu Z, et al. Angiotensin II receptor type 1 autoantibodies promote endothelial microparticles formation through activating p38 MAPK pathway. Journal of hypertension. 2014;32:762-770.
30. Fan XY, Chen B, Lu ZS, Jiang ZF, Zhang SQ. Poly-L-Arginine Acts Synergistically with LPS to Promote the Release of IL-6 and IL-8 via p38/ERK Signaling Pathways in NCI-H292 Cells. Inflammation. 2015;39:47-53
31. Chirinos JA, Zambrano JP, Virani SS, et al. Correlation between apoptotic endothelial microparticles and serum interleukin-6 and C-reactive protein in healthy men. The American journal of cardiology. 2005;95:1258-1260.
32. Cui Y, Zheng L, Jiang M, et al. Circulating microparticles in patients with coronary heart disease and its correlation with interleukin-6 and C-reactive protein. Molecular biology reports. 2013;40:6437-6442.
